# Africa 2010 in retrospect: hits and misses

**Published:** 2010-12-26

**Authors:** Robert Davis

**Affiliations:** 1 The American Red Cross, Nairobi, Kenya

**Keywords:** Millennium development goals, Africa, Public Health, measles, malaria, HIV/AIDS

## Abstract

**About the author:**

Robert Davis works for the American Red Cross as health and measles delegate, based in Nairobi, Kenya. A 1978 graduate of the Johns Hopkins University, he has just started his 25^th^ year in Africa, where he has worked with WHO, UNICEF, the International Rescue Committee, and Save the Children Fund. The views expressed in this editorial are his own.

## Editorial

As we see 2010 out the door and greet 2011, Africa has, amid many continuing concerns, much to be proud of in public health.

**The millennium development goals**

The 2010 United Nations review of progress towards the Millennium Development Goals (MDGs) showed a decline of under-five mortality in sub- Saharan Africa from 184 to 144 per1000 live births between 1990 and 2008. North Africa, mostly middle income, made better progress, with declines from 80 to 29 per 1000 over the same time period. At present rates of progress, most countries of sub-Saharan Africa are likely to fall short of MDG 4 [1]. The 2008 data did not fully reflect the impact of existing malaria interventions, much less the introduction of new vaccines for rotavirus and pneumococcal disease, still pending in most countries. Birth spacing, the best predictor of child survival, is not yet widely practiced in most African countries, as shown by successive Demographic and Health Surveys.

Most countries still lack high quality data on trends in maternal mortality. Process indicators in such areas as proportion of pregnant women getting at least 4 prenatal consultations and those having supervised deliveries were generally favorable, but more for Northern than for sub-Saharan Africa. The most cost-effective interventions against maternal mortality (late marriage, longer birth intervals, and higher contraceptive prevalence) are not yet a high priority in most African countries.

**HIV/AIDS**

According to the Joint United Nations Programme on HIV/AIDS (UNAIDS), "investments made to date in the AIDS response were seen to be bearing fruit as the rate of new infections stabilised or decreased by more than 25% in at least 56 countries around the world, including 34 countries in sub-Saharan Africa. . . In addition, more than 5 million people were estimated to be receiving antiretroviral treatment–a scale up of 30% in just one year. . . .However, 2010 was also a year in which for the first time resources for the AIDS response did not increase, with donor disbursements lower in 2009 than in 2008. This disappointing news came at a time when demand is continuing to outstrip supply in the AIDS response. For every one person who starts antiretroviral treatment a further two become infected with the virus [2]."

The year saw slow expansion of access to antiretrovirals, and scale-up of male circumcision in countries most likely to benefit from it. June saw the publication of an update on male circumcision in 13 priority countries [3].

Rwanda, which has so far circumcised youth and young adults, published evidence this year in favor of a more cost-effective approach aimed at neonates [4].

What is driving the pandemic? War and poverty are not causative; in fact, high income predicts high seroprevalence. Why is HIV declining in some places? If seroprevalence declines in Uganda and Zimbabwe were better understood, those could lead to adoption of their best practices in other countries of the continent.

**Malaria**

Reviewing progress against malaria at year's end, The World Health Organization (WHO) Director-General Margaret Chan pointed to the expansion of ACT and long life bednets in endemic countries of the continent [5].

By 2010, an estimated 578 million Africans had received malaria bednets. These and other data appeared in the World Malaria Report [6]. Page number not for citation purposes 3 While malaria surveillance remains a concern, survey data from several countries pointed to declines in morbidity and mortality. Writing in the Malaria Journal, Steketee and Campbell stated 'national malaria programme scale-up has achieved substantial impact across a growing array of African countries. The reductions in child mortality are consistent with or even greater than the estimated 20% reduction in all-cause child mortality predicted from the controlled trials of ITNs. In 2010, hundreds of thousands of African children will not die of malaria because of recent national investments" [7].

Just over the horizon loom the spectres of insecticide resistance and drug resistance. Artemisinin monotherapy continues, assisted by lax regulatory policies of producing and importing countries, the latter most likely to see malaria drug resistance in the current decade, with no obvious fall-back antimalarials once artemisinin resistance is established. A stroke of the regulatory pen would suffice to stop importation of inappropriate antimalarials, but the political will is lacking in many countries.

**Vaccine preventable diseases**

The Expanded Program on immunization (EPI), the continent's largest public health programme, saw a number of successes and a few setbacks in 2010. It was a generally favorable year for polio eradication. By Week 51 of 2010, Nigeria had registered 13 wild poliovirus cases, against 388 in 2009. Widespread use of monovalent and bivalent OPV led to interruption of transmission in West African reintroduction countries. At year's end, hundreds of AFP cases with a broad age distribution, including wild poliovirus cases, cropped up in polio free Congo/Brazzaville, probably originating from neighboring Angola. Polio free Uganda saw one case, genetically linked to Kenya's 2009 outbreak. All these countries, as well as Chad, are running mop-up campaigns using monovalent and bivalent vaccines [8].

Measles control moved slowly towards measles pre-elimination in 2010. Many southern African countries, the cradle of elimination efforts 10 years ago, saw persistent measles outbreaks. Outside Africa, WHO organized a meeting in Washington on the feasibility of measles eradication [9], a topic which came up later in the year at the meeting of the Strategic Advisory Group of Experts in Geneva. By year's end the future of measles eradication was less a question of whether than when

With 45 of WHO African region's (WHO/AFRO) 46 member states giving hepatitis B (hep B) vaccination, WHO/AFRO commissioned a position paper on viral hepatitis, with special attention to better surveillance of hepatitis B. The virus is, in theory, eradicable, but the long term carrier state of the virus in human subjects means that this goal is many decades in the future. A few European holdouts have not yet introduced the hep B vaccine, and two recipients of GAVI funding for hep B will "graduate" from GAVI in 2014, requiring them to purchase the vaccine themselves with foreign exchange.

The GAVI Alliance (GAVI) funding to Africa [10] saw little significant expansion in 2010, and by year's end few countries had launched pneumococcal and rotavirus vaccines. A vaccine donor's meeting will precede the next GAVI Board meeting in 2011.

The Sabin Vaccine Institute of Washington continued its work on the Expanded Programme on Immunization (EPI) financing in Africa, organizing meetings of parliamentarians and civil society leaders to advocate for greater government spending on vaccination. Meanwhile, several measles vaccination campaigns fell short of the benchmark of at least 50 percent local financing for measles Supplemental Immunization Activities (SIA) operations costs. At year's end, in-country financing of EPI remained, in all but a few middle income countries, more in posse than in esse, with health financing at well under 10% of total government expenditures in all but a few countries.

December saw the launching, in three countries, of the second generation vaccine against meningococcal meningitis. Expansion to all countries of the meningitis belt will require more funding, national and international.

**Demography**

UN-Habitat's 2010 report on African cities underlined the most notable demographic trend on the continent: the move to town from country, with the rise of African mega-cities of over 5 million people [11]. These bring new challenges to the health services, especially extension of basic health services to underserved slums, many lacking legal recognition. Except in urban areas, Africa is seeing very slow declines in fertility, with most of the world's high fertility countries in Africa [12]. Why, with the decline in under-five death rates, has Africa not yet seen the demographic transition from high death rates and high birth rates to declining death rates and declining births? Is it from lack of demand, or lack of supply?

**Partner perspectives**

This year saw the arrival at UNICEF headquarters of Anthony Lake, a new Executive Director looking to speed the move towards the health MDGs using equity based planning. Expect to read more and more UNICEF analyses of health indices based on gender and quintile analyses.

In a candid December report to African health leaders in Ouagadougou, WHO/AFRO reviewed progress against targets and laid down the following strategic directions for WHO: Partnership and resource mobilization, Health systems, PHC and community participation, Improved access to services to reduce maternal and child mortality, scale-up of interventions against AIDS, TB and Malaria, reducing the burden of diseases, preventing epidemics and reducing the risk of emerging public health threats, and working beyond the health sector to address health determinants.

**Hits and misses**

Despite gains on many fronts, the year saw little progress on several fronts: 1) Only three countries (Liberia, Rwanda and Zambia) reached the benchmark of 15 percent of government expenditure spent in the health sector (World Health Statistics 2010); 2) Few efforts to defuse the time bomb of tobacco associated mortality; 3) Underinvestment in neglected tropical diseases, despite local gains against guinea worm and river blindness; 4) Uneven progress in nationwide efforts at building health systems (Ethiopia's Health Expansion Project was a notable exception); 5) Insufficient investment in female education, which underlies, supports and predicts both MDG 4 and MDG 5. For every 100 primary schoolboys in sub-Saharan Africa, there are only 91 schoolgirls. In secondary school, the ratio is 100 boys to 79 girls. Seven African countries have abolished school fees, but net primary school enrolment rates in sub-Saharan Africa were only 76 %, according to the 2010 UN's MDG report [13]. Despite climbs in enrolment, sub-Saharan Africa still accounts for almost half the world's out of school children.

**The advocacy gap**

Last but not least, Africa has not been very good at putting its best foot forward. The best advocacy items on Africa that I saw this year came from Hans Rosling of the Karolinska Institute of Stockholm [14,15]. The diversity of Africa, with Mauritius and Sierra Leone at polar opposites, is only comprehensible when one gets away from regional averages. Hans Rosling's quintile presentations on Uganda and South Africa show the need to present and use existing databases.

African health ministries are notable by their low profiles and their reluctance to place their successes at center stage. Internal and external resources are not attracted by pictures of malnourished children, but by placing quantified successes in prominent display. When will we see African governments making their first Gapminder presentations [16]?

## Conflicts of interest

The author declared no conflicts of interest
